# Transcatheter closure of multiple ventricular septal ruptures after acute myocardial infarction: a case report highlighting the role of 3D transthoracic echocardiography

**DOI:** 10.1186/s43044-024-00601-3

**Published:** 2025-01-07

**Authors:** Tengku Winda Ardini, Yuke Sarastri, Joy Wulansari Purba, Yasdika Imam Taufik, Suci Asriri, Ali Nafiah Nasution

**Affiliations:** 1 Cardiovascular Department, Adam Malik General Hospital, Medan, Indonesia; 2 Department of Cardiology and Vascular Medicine, Faculty of Medicine, Universitas Sumatera Utara, Medan, Indonesia

**Keywords:** Multiple ventricular septal ruptures, Transcatheter closure, 3D echocardiography, Case report

## Abstract

**Background:**

Post-infarct ventricular septal rupture (PI-VSR) is a rare complication of acute myocardial infarction (AMI) but has very serious implications. Managing PI-VSR using transcatheter closure (TCC) presents varying challenges depending on the patient’s condition. The aim of this study is to present a highly challenging case of multiple VSRs as a complication of AMI.

Case presentation: A 59-year-old male was admitted with symptoms of shortness of breath, dyspnea on exertion, orthopnea, and swelling of the lower extremities. He had typical chest pain related to infarction 2 weeks before his admission. On electrocardiogram (ECG) examination, evidence of an old myocardial infarction in the infero-antero-lateral regions was seen. Echocardiography showed mild mitral and tricuspid regularities. The left ventricular (LV) systolic function was mildly compromised, with a global ejection fraction of 44%. There was also a left-to-right VSR shunt in the apical region of the LV. Multiple defects as outlined by 3D transthoracic echocardiography (TTE)—the largest measuring 17 mm. Given the high risks of open-heart surgery, a percutaneous closure of the VSR was carried out using a 21 mm atrial septal defect (ASD) occluder. The device was satisfactorily placed, and there was an improvement in the clinical condition of the patient. He was discharged after his 8-day stay in the hospital.

**Conclusion:**

Our study emphasizes that echocardiography with 3D imaging provides a more detailed view of the size and shape of the rupture and serves as a valuable modality for guiding the percutaneous transcatheter VSR closure procedure.

## Background

Acute myocardial infarction (AMI) continues to be a significant global health problem with an incidence of nearly three million patients worldwide [1]. Study has also shown that AMI tends to be more common with advancing age, with a prevalence of 3.8% in people under the age of 60 years, and as high as 9.5% among those over the age of 60 years [2] In addition, the mortality of AMI is high in that more than a million deaths are reported annually. Anterior myocardial infarction (60%) is considered more frequently than inferior chamber location, and there is a tendency to lead to irreversible damages of the myocardium due to the prolonged period without oxygen [1]. There are many complications of AMI, and one includes ventricular septal rupture (VSR). Post — Infarct ventricular septal rupture (PI-VSR) is one of the very rare, but fatal complication after AMI. The incidence of PI-VSR before the era of reperfusion therapy was 1% to 2%, but this decreased markedly (0.17%—0.31%) after the introduction of reperfusion therapy. 3

Transthoracic echocardiography (TTE) represents the first-line diagnostic approach in PI-VSR. It is a necessary diagnostic tool for detailed identification of the anatomic location and size of the defect because focal interruptions in the contour of the interventricular septum (IVS) can be depicted along with left-to-right shunts with color Doppler. By contrast, additional value may be achieved by transesophageal echocardiography (TEE) both preoperatively and intraoperatively if the transthoracic windows are poor. However, TTE is often less sensitive about the size of the defect due to the ultrasonic beam not being oriented coaxially with the rupture. Moreover, a more detailed qualitative and quantitative assessment of the defect is possible with 3D echocardiography compared with 2D echocardiography [4]. In the management of PI-VSR, ACCF/AHA recommend urgent surgical intervention independent of hemodynamic status of the patient [5]. This surgical intervention is, therefore, associated with the serious risks of bleeding from fragile myocardial tissue and residual shunts after surgery. Some studies suggest, based on such serious risks, that surgical intervention should be postponed when the patient’s condition is unstable [6, 7]. Moreover, the 2017 ESC guidelines for the management of AMI recommend that surgical procedures for PI-VSR patients may be postponed until the condition has stabilized after initial medical management [8]. In this regard, TTE-guided transcatheter device-based VSR closure has been forwarded as an alternative and promising technique; in particular, more detailed and accurate visualization can be obtained. This study, therefore, aims to report a case of PI-VSR and the utility of 3D TTE in the accurate delineation of the shape, number, and size of such defects for occlusion.

## Case presentation

We received a 59-year-old male patient who presented to our hospital with complaints of worsening shortness of breath over the 3 days prior to admission. The shortness of breath was exacerbated by physical activity and changes in position. Additionally, the patient experienced bilateral lower extremity edema. These symptoms led to the patient being hospitalized at a rural hospital for 3 days. He was subsequently referred to our hospital due to a worsening hemodynamic condition and a diagnosis of anterolateral ST-elevation myocardial infarction (STEMI). On physical examination, the patient was alert and well-oriented, with a blood pressure of 98/68 mmHg, a heart rate of 107 bpm, a respiratory rate of 20 breaths per minute, and an oxygen saturation of 93% on room air, which improved to 96% with the use of a nasal cannula at a flow rate of 4 L/min. We also detected a grade 4 pan-systolic murmur at the lower left sternal border.

We subsequently performed several diagnostic examinations on the patient. The electrocardiogram (ECG) showed sinus tachycardia with a QRS frequency of 115 bpm, left atrial deviation, left atrial enlargement, and signs of an old anterolateral myocardial infarction. The chest X-ray revealed cardiomegaly with a cardiothoracic ratio of 62%, aortic elongation, pulmonary congestion, and bilateral lung infiltration. Additionally, laboratory tests showed abnormalities such as leukocytosis (15,720/mm^3^), impaired renal function (urea 111 mg/dL and creatinine 2.96 mg/dL), and elevated troponin I levels (14.6 ng/mL).

**Fig. 1:**
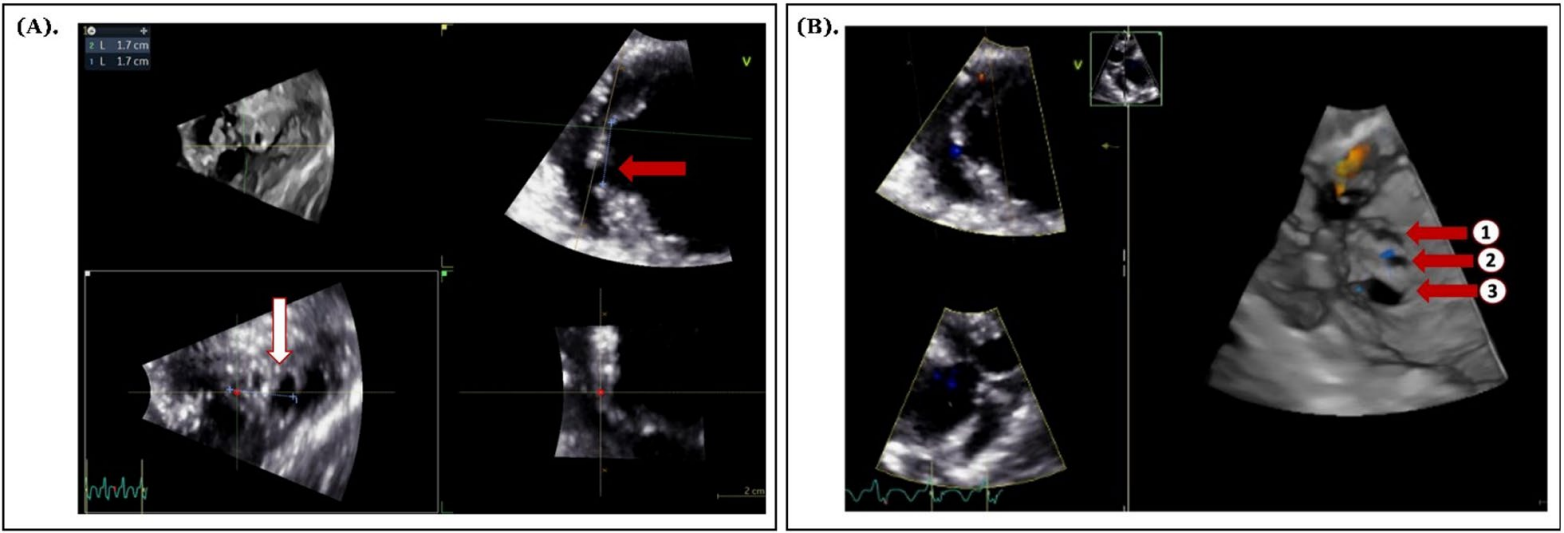
3D TTE imaging of VSR. **A** The 3D TTE image reveals multiple VSR defects located in the apical region of the left ventricle, with the largest defect measuring 17 mm, indicated by red and white arrows. **B** An En Face view from the 3D TTE modality shows the shape and clear delineation of the three VSR defects, highlighted by three red arrows

The patient also underwent an echocardiography examination, which showed a left ventricular ejection fraction (LVEF) of 48% (biplane), grade II diastolic dysfunction with elevated left atrial pressure (E/A ratio: 0.78; E/e′: 15.59), and moderate functional tricuspid regurgitation with a high probability of pulmonary hypertension (TR Vmax: 4.04 m/s; TR PG: 65.44 mmHg). Additionally, we found a rupture of the interventricular septum (IVS) in the apical left ventricle (LV), with the longest diameter measuring 17 mm and a left-to-right shunt (pressure gradient: 54.09 mmHg). During the TTE examination using 3D imaging, we also identified multiple defects in the apical region of the LV (Figs. 1A and 1B). Furthermore, we found three defects of varying sizes, with a total length reaching 17 mm. Ultimately, we determined that the patient’s diagnosis was multiple VSRs as a complication of AMI.

Regarding the management of this patient, since the patient had a high surgical risk, we planned a transcatheter closure (TCC) for the PI-VSR guided by 3D TTE. We first evaluated the shape and size of the defect. Based on this evaluation, we decided to use a 21 mm atrial septal device (ASD) occluder, which we deemed sufficient to close all the VSR defects. This decision was based on our finding that the largest defect measured 17 mm. Additionally, the 3D echocardiography examination showed an adequate distance from the defect to the apical wall of the LV, indicating that an ASD occluder with wings measuring 6–7 mm would be suitable for closing the existing defect.

**Fig. 2:**
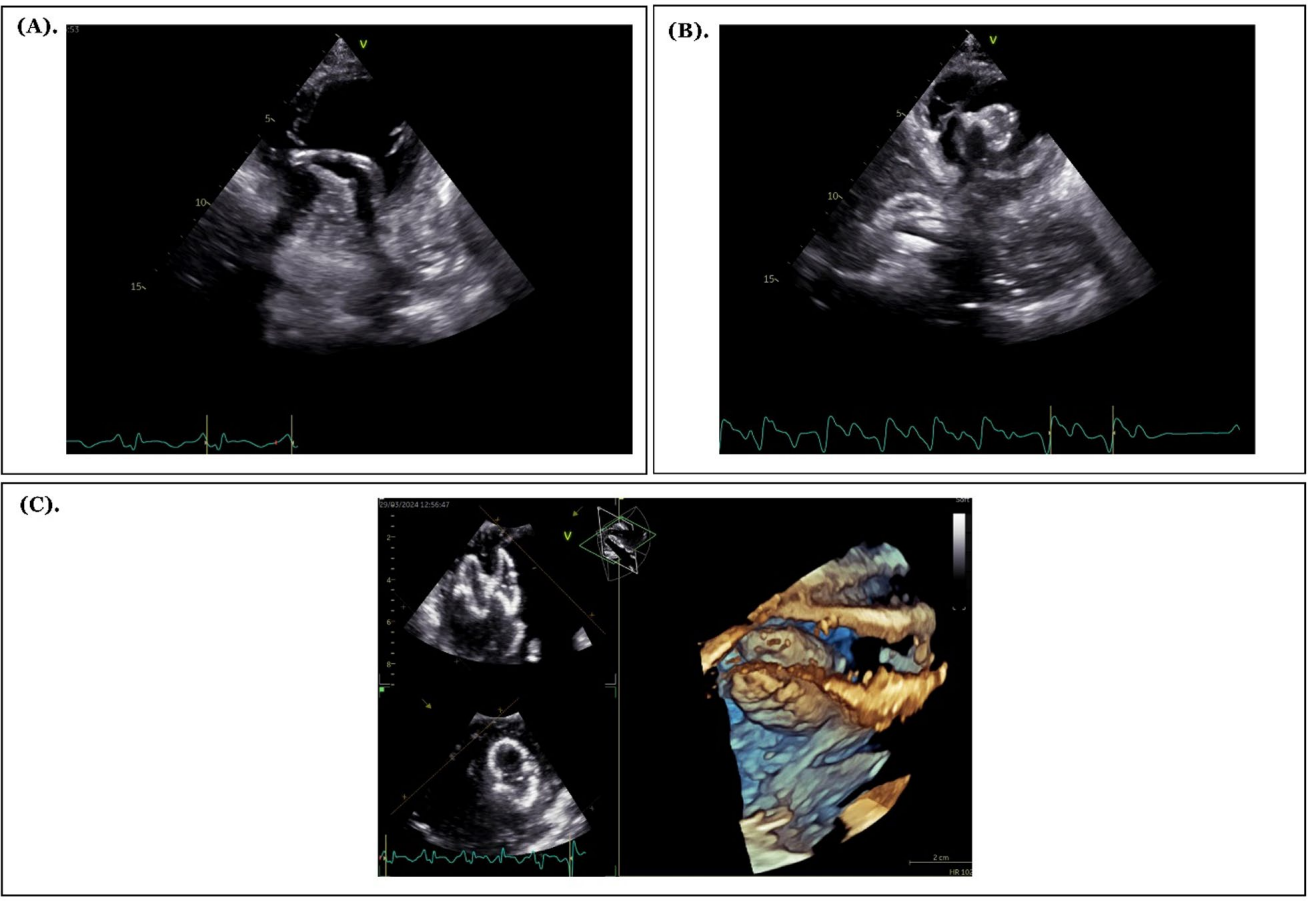
2D and 3D TTE imaging during and after transcatheter closure of VSR. **A** The 2D TTE image displays the catheter crossing from the RV to the LV through the VSR defect. **B** The 2D TTE image shows the ASD device being deployed in the LV and then pulled into the RV to cover the VSR defect. **C** The 3D TTE evaluation after device deployment confirms that the device is well positioned over the VSR defect, with mild central leakage observed

In the management of this patient, we positioned the intra-aortic balloon pump (IABP) in the third intercostal space (ICS) with a frequency setting of 1:1 and trigger pressure augmentation of 100%. Through coronary angiography, we found total stenosis in the proximal left anterior descending artery (LAD) and several 20% stenoses in the mid-right coronary artery. Additionally, left ventriculography identified contrast flow from the LV to the right ventricle (RV) through a septal defect. We then proceeded with placing a wire and catheter from the RV to the LV through the defect, guided by fluoroscopy and assisted by TTE (Fig. 2A). Subsequently, we inserted an ASD occluder device through the defect (Fig. 2B). Our evaluation showed that the device was well-seated on the defect, with minimal central residual flow (Fig. 2C). After the TCC procedure, the patient’s hemodynamic condition remained stable. To closely monitor the patient, we transferred them to the cardiovascular care Uuit (CVCU). The patient was eventually discharged from the hospital on the 8th day.

## Discussion

Our study reported a patient with AMI who experienced complications in the form of multiple PI-VSRs, detected through 3D TTE. This complication was successfully managed with closure using an ASD occluder device on the 20th day after the onset of acute coronary syndrome (ACS). Following the closure procedure, the patient’s clinical condition improved with stable hemodynamics. Several similar case reports have been documented in Australia [9], China 10], and India [11], all reporting successful closure of VSRs due to complications from AMI. In Indonesia, this is only the second case report of its kind. A previous case in Medan demonstrated that an IABP could benefit a patient with intractable heart failure due to MI of undetermined onset, complicated by VSR [12]. By utilizing 3D TTE, our study adds to the range of approaches for managing patients with VSR resulting from AMI complications.

The VSR complication due to AMI reported in this study has a complex mechanism. In patients with AMI, coagulation in the necrotic area begins within the first 24 h. During this process, neutrophils attack the infarcted tissue, causing apoptosis and releasing lytic enzymes that destroy the necrotic myocardial tissue [13, 14]. This leads to the characteristic pathological features of VSR: necrosis of ischemic myocardial tissue, followed by neutrophilic infiltration, tissue thinning, and eventual septal rupture [10]. The anatomical location of the rupture depends on the affected arterial territory. Anterior infarction is more likely to cause defects in the apical septum, whereas non-anterior infarction typically results in defects in the basal septum. Defects in the basal septum can potentially involve the posterior wall, adding complexity to the closure procedure [4]. VSR as a complication of anterior myocardial infarction accounts for about 60% of cases, while the remaining 40% involve inferior myocardial infarction [10]

Regarding the timing of the rupture complication in this patient, the data from this study align with the theoretical prediction of rupture timing. In this case, a murmur was detected three days after the onset of ACS. Theoretically, early rupture occurs in infarctions with large intramural hematomas that dissect the surrounding tissue. If the patient survives this condition, fibrotic tissue remodeling will begin after several weeks. Furthermore, rupture usually occurs within the 1st week following myocardial infarction. More specifically, ruptures often occur during two peak time periods: within the first 24 h and between 3–5 days post-infarction. Ruptures rarely extend beyond 2 weeks after the infarction [13, 15]

In this patient, the detected defects were multiple and located in the apical area. This condition posed a particular challenge in determining the success of the closure. VSR defects are categorized as simple or complex, based on their pathway and relationship to surrounding structures. Simple defects involve direct communication between the LV and RV that is entirely within the septum. In contrast, complex defects can follow a serpiginous pathway between the ventricles and may extend beyond the septum. Furthermore, complex defects vary in size, ranging from a few millimeters to several centimeters. Additionally, the shape and size of complex defects can change throughout the cardiac cycle, with paradoxical enlargement during systole when shunt volume increases. This phenomenon is caused by the non-contractile nature of the septal muscle. [4]

In this patient, since TEE was inadequate for visualizing the apical septum, we proceeded with 3D TTE only. The procedure we performed was consistent with the literature. According to the literature, TTE or TEE at the patient’s bedside is the diagnostic modality of choice for early detection and therapeutic guidance in patients suspected of having VSR. PI-VSR often has a complex structure, with multiple orifices and serpiginous tunnels. Due to these characteristics, visualization and measurement of the defect may be less optimal when using only 2D TTE. On the other hand, 3D echocardiography offers better delineation of the size and shape of the rupture [12]. In this patient, because the defect was located in the apical area, TTE provided a clearer view compared to TEE. Some advantages of 3D echocardiography include improved identification of various anatomical features in different types of VSR. Additionally, real-time 3D echocardiography (RT3DE) provides real-time imaging and measurement capabilities, which, in turn, enhances the utility of this modality. Furthermore, 3D echocardiography allows for accurate assessment of VSR, whether the defect is circular or elliptical in shape, and can display dynamic features throughout the cardiac cycle. The en face view provided by 3D echocardiography allows for a detailed assessment of both the defect and surrounding tissue [16]. RT3DE can also accurately evaluate the size, shape, and distance of the defect from the apical wall. This is crucial for selecting the appropriate type and size of the occlusion device. Additionally, RT3DE serves as an important guide during percutaneous transcatheter PI-VSR closure procedures. [17]

In this patient, we performed TCC 2 weeks after the MI event. Major guidelines recommend surgical repair for patients who develop VSR following AMI, with TCC as a secondary option [5, 8]. However, with the advancement of medical knowledge, VSR closure can be achieved using percutaneous TCC. This approach is particularly relevant for patients who are critically ill or have multiple comorbidities [6, 7]. The advantage of this procedure over surgical repair is that it offers faster hemodynamic stabilization. Additionally, TCC can serve as a definitive treatment for patients who are hemodynamically stable with anatomically suitable defects, such as defect sizes less than 15 mm, and in cases of subacute to chronic post-MI VSR, or more than 3.5 weeks. [18] Regarding the timing of intervention in this case, it has been suggested that the prognosis for VSR patients tends to be poor if the intervention is performed early, while the prognosis improves if the procedure is delayed for 2 weeks or more. This is due to several factors, including VSR maturation, recovery of myocardial function, establishment of collateral blood supply, and adaptation to hemodynamic changes. Furthermore, emergency surgical intervention often results in an increased risk of mortality due to unstable conditions [10]. This is supported by several studies indicating that delaying surgical intervention for 3–6 weeks, if hemodynamics allow, can lead to a better prognosis. This delay gives time for the infarcted myocardium to develop a strong scar, which in turn facilitates more effective surgical repair. [10, 19]

In the management of this patient, we used an IABP to mechanically reduce afterload. The primary principle of conservative management for PI-VSR is afterload reduction to improve left ventricular stroke volume by decreasing the left-to-right shunt, either pharmacologically or mechanically [20]. Mechanical afterload reduction can be achieved through the use of an IABP. It is well known that the primary cause of death in patients with AMI complicated by VSR is pump failure, which leads to hemodynamic instability. Several factors that contribute to increased mortality in VSR patients include cardiogenic shock, early-onset VSR, and reduced left ventricular function. Additionally, other factors that play a role in mortality in VSR patients are multi-organ failure, ventricular arrhythmias, and cerebral infarction [21]. According to the SHOCK trial and GUSTO-I, the 30-day mortality rates for AMI with VSR were 87% and 74%, respectively [22, 23]. Furthermore, inferior-basilar septal ruptures are associated with a 1.73 times higher risk of mortality compared to anterior-apical defects, mainly due to more challenging intraoperative access. Considering the timing of intervention, the mortality of PI-VSR patients varies significantly depending on the timing of surgical intervention. Patients who underwent surgery within 7 days after diagnosis had a mortality rate of 54.1%. However, if surgery was delayed for more than 21 days, the mortality rate decreased to 10% [24].

## Conclusion

Rapid identification and reperfusion in patients with AMI are the most effective strategies to prevent post-MI structural complications. Although rare, PI-VSR is a life-threatening complication with a high mortality rate that requires timely repair. In this case, we successfully performed TCC of multiple PI-VSRs guided by 3D TTE. This modality allowed for accurate evaluation of the number, shape, and size of the defects, facilitating precise device selection and optimal guidance during the closure procedure, ultimately leading to improved clinical outcomes for the patient.

## Data Availability

No datasets were generated or analyzed during the current study.

## Abbreviations

ACCF: American College of Cardiology Foundation
ACS: Acute coronary syndrome
AHA: American Heart Association
AMI: Acute myocardial infarction
ASD: Atrial septal defect
CVCU: Cardiovascular care unit
ECG: Electrocardiogram
ESC: European Society of Cardiology
GUSTO-I: Global Utilization of Streptokinase and TPA for Occluded Coronary Arteries
IABP: Intra-aortic balloon pump
ICS: Intercostal space
IVS: Interventricular septum
LV: Left ventricle
LVEF: Left ventricular ejection fraction
MI: Myocardial infarction
PI-VSR: Post-infarction ventricular septal rupture
RT3DE: Real-time 3D echocardiography
RV: Right ventricle
SHOCK: Should We Emergently Revascularize Occluded Coronaries in Cardiogenic Shock
STEMI: ST – Elevation myocardial infarction
TCC: Transcatheter closure
TR PG: Tricuspid regurgitation peak gradient
TR VMAXTRICUSPID: Regurgitation velocity max
TTE: Transthoracic echocardiography
VSR: Ventricular septal rupture
